# Failure on all fronts: general dental practitioners’ views on promoting oral health in high caries risk children- a qualitative study

**DOI:** 10.1186/s12903-015-0032-8

**Published:** 2015-04-09

**Authors:** Ahmad K Aljafari, Jennifer Elizabeth Gallagher, Marie Therese Hosey

**Affiliations:** Division of Population and Patient Health, King’s College London Dental institute, Bessemer Road, London, SE5 9RS UK

**Keywords:** Early childhood caries, Dental prevention, Oral health promotion, Primary dental care, Qualitative research, High caries risk

## Abstract

**Background:**

Despite overall improvements in oral health, a large number of children in United Kingdom (UK) are affected by dental caries; and the implementation of oral health promotion in some families remains a challenge. As such, children from those families suffer high caries rates, and are frequently referred for tooth extraction under General Anaesthesia (GA), one of the commonest reasons for paediatric hospital admissions. The aim of this investigation is to explore referring primary care General Dental Practitioners’ (GDPs) views and experiences in trying to promote better oral health for those children.

**Method:**

A qualitative study, utilizing face-to-face, semi-structured interviews with GDPs in three London boroughs who refer children for extraction of decayed teeth under GA selected based on referral rate. Qualitative Framework Analysis was used to present the results.

**Results:**

Eighteen GDPs (56% male) were interviewed: average age 42 years (range: 26–73 years). informants reported challenges to promotion of oral health categorised as: (1) child’s young age, poor cooperation, and high treatment need; (2) parental skills to face up to modern day challenges and poor attitudes towards good oral health (3); social inequality, exclusion and cultural barriers in immigrant families; (4) National Health Services (NHS) primary care practice remuneration, constraints and training; (5) inadequate secondary care communication and engagement; and (6) failure in establishing national policy to grasp the width and depth of the problem.

**Conclusion:**

GDPs feel frustrated and isolated in their efforts to promote oral health in those children. These findings suggest difficult challenges on all fronts. Reform of preventive dentistry funding and delivery, as well as a multiagency multidimensional approach that is mindful of the social determinants of children’s oral health and barriers to application of oral and wider health initiatives are needed to address this important public health issue.

## Background

Dental caries is a disease that ideally is completely preventable. Yet, caries in early childhood is a worldwide problem [1,2]. A significant proportion of children in England suffer from the disease [3,4]. More worryingly, many children, especially those from poorer socio-economic areas, end up requiring tooth extractions to manage the issue [5,6]. Indeed, tooth extraction, mainly under General Anaesthesia (GA), is the main reason for hospital admissions of 5–9 year old children [7]. Repeat treatments are frequent (20%-25% of cases) [8-10], in many instances due to failure in reducing risk and altering treatment patterns.

Children receiving dental extraction under GA are mostly considered to be of high caries risk as evident by their high treatment needs [9], yet focus on preventive dental care (any activity by which an individual avoids the development, progression and reoccurrence of an oral disease), and the wider concept of oral health promotion (any combination of oral health education and legal, fiscal, economic, environmental, organizational and technical interventions designed to facilitate the achievement of oral health and the prevention of disease) [11], continues to be inadequate. In a 2011 study, the majority (71%) of parents of children referred for tooth extraction under GA requested help in promoting oral health in their families. yet 61% had no plans for continuing dental care for their child. Only 45% indicated that they received advice on dose of fluoride in toothpaste and fewer still were offered fluoride varnish (8%) or fissure sealants (10%) [10]. A previous qualitative study by the present authors supported those findings and further revealed that parental oral health knowledge, parenting skills, as well as previous advice received are all relevant factors in the oral health of those children [12].

The NHS provides free dental care for all children in the UK. Parents are advised to use a local GDP in a primary care setting. In cases where specialist or hospital care is needed, the child is generally referred but some will present in an emergency at dental hospitals. As such, GDPs provide routine dental care for children and play a vital role in promoting oral health. An evidence based toolkit to inform appropriate preventive care has been available in the United Kingdom (UK) since 2007, with the latest edition being published recently [13]. However, it’s been reported that GDPS are struggling to be thorough and consistent in their application [14], and have indicated that its delivery is difficult when adequate resources or staff support aren’t available [15].

It is important to realise that once a child develops dental caries they are more likely to develop more caries and more likely to suffer pain and sepsis [16,17]. Despite the ongoing debate on how GDPs can best manage caries in young children, one thing is clear: that prevention is of paramount importance and a change in the approach to preventive care delivery and oral health promotion in this cohort of children is needed. The Steele Report in 2009 stressed the importance of reforming the way preventive care is provided under the National Health Services (NHS) [18]. Other authors stressed the need to design intensive preventive interventions for children suffering from the disease and assess their efficiency [16].

The aim of this investigation was to explore the GDPs’ experience and views in regards to providing preventive dental care for high caries risk children, as defined by those referred for tooth extraction due to caries, as well as explore their opinion on what is needed to promote oral health in that cohort.

## Methods

This study involved a qualitative investigation utilizing semi-structured interviews. It was granted ethical approval by King’s College London Biomedical Sciences, Dentistry, Medicine and Natural & Mathematical Sciences research ethics committee (Reference number: BDM/12/13-34). Information regarding the research team can be found in the (authors’ information) section.

The targeted informants in this study were GDPs working in the referral area for King’s College Hospital (KCH), which includes the south London Boroughs of Lambeth, Southwark and Lewisham (LSL), provided they had referred children for management of caries under general anaesthesia. These boroughs are some of the most highly deprived in England, ranking 15^th^, 17^th^ and 24^th^ respectively in deprivation in 2010 [19]. They are also known to be culturally diverse, containing people from various ethnic minorities and immigrant backgrounds. The National Census in 2011 reported that almost 40% of adult residents in those areas were born outside the UK [20]. The rate of child attendance for dental care is poor, and highly associated with social deprivation in those areas [21].

Purposive sampling, based on GA referral rates to hospital, was used. A list of general dental practices that had referred children to King’s College Hospital from March 2011 to March 2012 was obtained and practices were sorted into three categories:

1. High referrers: 15+ referrals a year (7 practices).
2. Medium referrers: 5–14 referrals a year (36 practices).
3. Low referrers: <4 referrals a year (123 practices).

Invitation letters and information leaflets that detailed the aims and design of this research project were sent by post to practices from all three categories. Our aim was to collect the opinions of dentists of various ages, work experience, gender, and referral rate. Researcher (AA) followed the postal invitation with a phone call one week later to inquire about willingness to participate. He then arranged to visit those who agreed to take part to perform the interview face to face in the informant’s own dental practice. Following a brief introduction, there was an opportunity for clarification and questions prior to obtaining written consent, and commencing the interview.

The interview schedule included open-ended questions and was divided into five discussion topics: (i) informants’ basic information, (ii) experience with referral of children for management of caries under general anaesthesia, (iii) preventive dental care provided for those children, (iv) views on the hospital service, and (v) views on promoting the oral health of those children.

The design of the interview was re-assessed by the researchers after the first five interviews. At this stage, a further question regarding the informant’s familiarity with England’s preventive dentistry guideline (Delivering Better Oral Health: An evidence-based toolkit for prevention) [13] was added.

All interviews were audio recorded and transcribed verbatim. All data were anonymised prior to analysis; informants are identified only by their referral rate, experience and gender. Descriptive statistics were used to present the demographics of informants. Framework Analysis, a rigorous approach for ordering, synthesising and presenting qualitative data [22], was used to report on the interviews. Microsoft Office Excel was used as the platform for analysis. An analytical framework was informed by relevant literature, interview schedule and emerging text of the interviews. Steps of analysis included familiarisation with raw data, development of a thematic index, theme refinement, charting into the relevant part of the framework and finally developing explanations and looking for applications to wider theory. The research team has met regularly during data collection and analysis to discuss the process of coding and theme assignment and any disagreements were solved by discussion. The consolidated criteria for reporting qualitative research (COREQ) [23] were used as a guide to ensure quality.

## Results

Data collection took place between February and April, 2013. Fifty one dental practices were invited to participate. Those included all six high referral practices, 14 medium referral practices, and 31 low referral practices in LSL. Invitations were sent with the aim of achieving balance and representation across the groups. Establishing direct communication with potential informants in many cases was challenging, due to their commitment to providing clinical treatment during working hours and unavailability outside of those hours.

In the course of the study, the researcher was able to make contact by phone with 25 dentists from 21 clinics. Eighteen dentists from 14 different practices agreed to take part and were subsequently interviewed. Most of those that refused, identified time constraints as the reason. Thematic saturation was reached; “Thematic saturation” occurs when the content of new interviews repeats that of previous interviews and is a common method of determining if sufficient data has been collected in qualitative research [24].

The average age of the informants was 42 years (42.3 years, Range: 26–73, SD: 13.8 years). Out of the 18 informants, 10 were male (55.6%). On average, they had 17 years of experience (17.2 years, SD: 13.5, Range: 2–43 years) as a dentist and 12 years (11.9 years, SD: 12.9, Range: 1–40 years) of experience practicing in their respective neighbourhood. Seven informants (39%) were principal dentists. Five (28%) were from high, six (33%) from medium, and seven (39%) from low referral practices.

**Figure 1:**
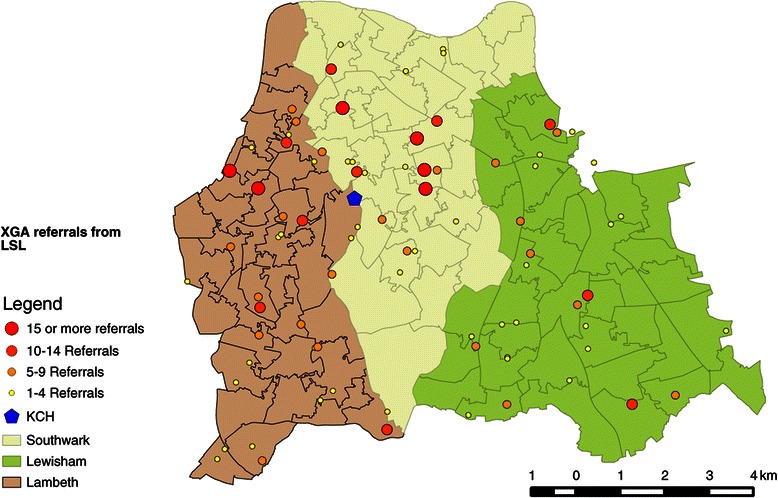
**Map of referrers for XGA from LSL (March 2011- March 2012).**

One thousand and two children underwent extraction of carious teeth under general anaesthesia at King’s College Hospital between March 2011 and March 2012. Seven hundred and fourteen (71%) of them were from the LSL Boroughs. Names of referrers of 307 children were recorded as missing on the hospital database, leaving 695 referred from 166 referring practices available. Eighty four of those practices were in LSL Boroughs; representing 79% of the total number of practices in this catchment [25]. The number of children referred by each practice ranged from 1 to 24. Figure 1 shows the location of all referring practices in LSL Boroughs.

**Figure 2:**
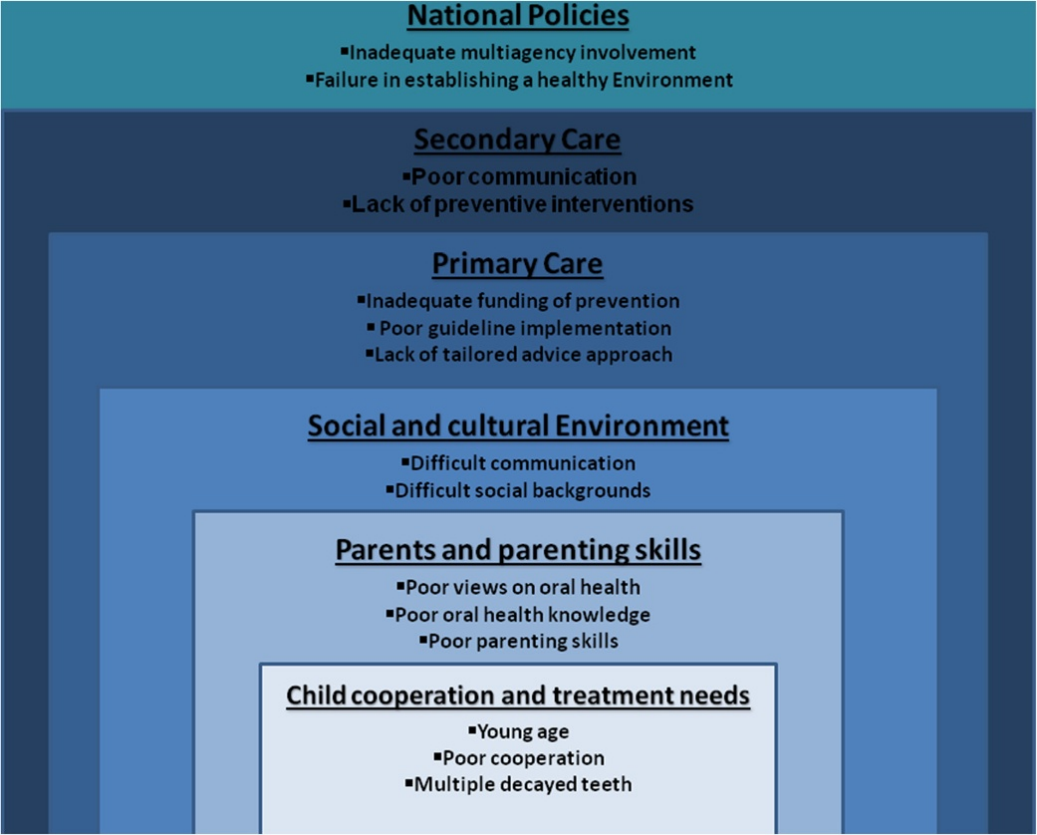
**GDP perceived challenges to promoting oral health of high risk children referred for GA tooth extraction (XGA).**

Analysis of qualitative data revealed that GDPs perceive challenges to the provision of preventive care and to the promotion of oral health amongst this cohort of children that can be attributed to every element involved in their oral health care: starting with the individual (child), and ending with wider public policy. These barriers can be categorised as follows: (1) child’s young age, poor cooperation, and high treatment need, (2) parental skills to face up to modern day challenges and poor attitudes towards good oral health (3) social inequality, exclusion and cultural barriers in immigrant families, (4) NHS primary care practice remuneration, constraints and training, (5) inadequate secondary care communication and engagement, (6) failure in establishing national policy to grasp the width and depth of the problem. [Figure 2] represents a summary of the results and displays the aforementioned challenges.

Following are the details of those challenges and a discussion of possible approaches for the future.

### Child’s young age, poor cooperation, and high treatment needs

Interviewees reported that children referred for treatment under general anaesthesia are usually of young age, poor cooperation, and present with multiple caries lesions. As one dentist explained about her referral criteria:

*“Their age, how decayed their teeth are and how cooperative they’re going to be with us.”* ^*P3, 29 YO, High referrer*^

These factors make the provision of preventive dental care, such as fluoride varnish application, appear time consuming. This limits the amount of preventive care provided to those children, as one dentist explained:

*“I mean like to actually prepare a child for fluoride treatment varnish and all that it does require quite a bit of time and it is not just open your mouth, you know they could be uncooperative.”* ^*P12, 57 YO, Low referrer*^

In addition, the late presentation of those children means they frequently present in pain. In informants’ view, this suggests that the families are less interested in preventive care. One dentist explained the issue:

*“A lot of them will be in pain and all they want to do is just get rid of that pain and they are happy.”* ^*P9, 26 YO, Medium referrer*^

Thus, informants perceived that the late presentation of young children, in pain, and with multiple decayed teeth, reduces the priority for oral health promotion and preventive care in the view of both the GDPs and parents.

### Parental skills to face up to modern day challenges and poor attitudes towards good oral health

Informants perceived that parents of this cohort of children have negative attitudes towards dental care and lack oral health knowledge. In addition, they felt that those parents display what they view as poor parenting practices. They expressed frustration with their infrequent dental attendance and felt that those families view dental appointments as “emergency services” only, leading to late presentation and mounting to neglect, as informants explained:

*“They just access you purely for emergencies and you begin to see that look you are just supervising neglect here so you might as well just succumb to their requests because the child is effectively being abused.”* ^*P13, 48 YO, Low referrer*^

*“The general scenario is that it is usually a neglected state, it is an emergency appointment and the families are just like passers-by.”* ^*P15, 34 YO, Low referrer*^

Informants believed that those parents see the GA pathway as perhaps the “easy way out”. Many reported that parents walk in specifically asking for their child to be referred for treatment under general anaesthesia:

*“…There are parents who will go to the practice and demand: I don’t want to be treated, I just want you to send me to the hospital, that’s what my other daughter did and that’s what my other son and it was one and they took it all out.”* ^*P13, 48 YO, Low referrer*^

Informants suggested that parental anxiety was a factor that might be contributing to this poor attitude towards dental attendance and care. They noted that parents avoid attending dental appointments themselves and appear to be transmitting their anxiety to their children:

*“It appears to be sometimes mothers are more scared than their kids so they just want everything to be done at the Hospital.”* ^*P2, 32 YO, High referrer*^

*“… They also teach them that it’s scary to come to the dentist, they are scared parents and the children learn this, the same behaviour, they don’t come to check-ups.”* ^*P14, 39 YO, Low referrer*^

Informants also reported that in many cases, families are not familiar with the concept of prevention of dental caries, especially when it comes to hidden sources of sugar and the use of fluoride:

*“They don’t consider any other source of sugars in the food and the drinks, like juices, fizzy drinks.”* ^*P6, 33 YO, High referrer*^

*“… They are not very well educated about caries and caries risk, and you know, nutrition or diet or fluoride, you know, at the onset on the teeth.”* ^*P18, 43 YO, Low referrer*^

However, even when oral health advice is given, informants felt that parents consistently fail to adhere to it. This leads them to believe that those families have poor attitudes towards the importance of oral health and poor parenting practices. As such, an undercurrent of despair and frustration can be felt talking to the informants, as they struggle to promote oral health in those families.

*“I tell you what, we sometimes tell them here and they walk out and their parents give them sweets, I’m like, hey I just told you! ‘Yeah, but he was a good boy’. Waste of time!”* ^*P7, 59 YO, Medium referrer*^

*“We can just say (Advice) but they don’t follow most of the time, they don’t follow and sometimes they come again and they say that was never told before”.* ^*P2, 32 YO, High referrer*^

### Social inequality, exclusion and cultural barriers in immigrant families

Informants reported that the social inequalities in oral health were obvious. They described a divide between children who were caries free, regular attendees that receive preventive care, and those with multiple caries lesions and poor attendance that do not receive the preventive care they desperately need:

*“You have two sets of patients, one absolutely perfect, and nothing to be done. They come in, Duraphat varnish, oral hygiene instructions, a clean-up, out. And other ones, gross, there’s nothing in-between.”* ^*P7, 59 YO, Medium referrer*^

Establishing rapport with parents was reported to be challenging sometimes, and this was seen as a hindrance to the delivery of oral health advice. It was interesting to note however that informants felt that establishing rapport with the children was easier:

*“We have a lot of people who come from difficult backgrounds in the family. Sometimes I actually cannot even make rapport with the parents so I would make rapport with the kid.”* ^*P15, 34 YO, Low referrer*^

In what might reflect an issue more local to the practices’ catchment area, many informants pointed out that children from immigrant families, usually attending the dentist for the first time, constitute a large portion of those referred for caries treatment under general anaesthesia:

*“I’ve been in [Location] for twenty three years … The individuals who do attend with a high caries incidence are those people who come from outside the UK.”* ^*P10, 49 YO, Medium referrer*^

*“Most of these people that I see with rampant decay are actually people coming from outside”* ^*P12, 57 YO, Low referrer.*^

*“The new patients in the practice tend to be new immigrants they tend to have higher caries experience.”* ^*P13, 48 YO, Low referrer*^

Informants generally felt that failure to reach immigrant families earlier, and to establish a regular pattern of dental attendance, is mainly due to difficulties in communication. However, those difficulties were not just limited to language, but also to cultural and social factors that they felt affect the parents understanding of the role of the general dentist.

*“The biggest block has always been communication for these people. So even when they’ve arrived here, knowing we have a full range of facilities, there’s a little bit of anxiety in, in going out and seeking help etc.”* ^*P10, 49 YO, Medium referrer*^

*“One thing I say, it’s generally the families where they don’t speak too much English, that’s where I notice a lot of the decay in the baby teeth and things like that.”* ^*P3, 29 YO, High referrer.*^

*“The trouble usually is the barrier is not language per se, it is attendance, because they don’t see the dental situation as a priority.”* ^*P13, 48 YO, Low referrer*^

### NHS primary care practice remuneration, constraints and training

Funding of preventive care in primary practice was noted as a major issue. There was a consensus between informants that the current NHS England remuneration system doesn’t provide enough support for preventive care for those children, and favours a treatment rather than prevention approach:

*“Well they said it rewards preventative treatment, we don’t think so”.* ^*P12, 57 YO, Low referrer*^

*“You are not going to be paid more if you bring a patient in three times a year and apply topical fluoride, but you will be paid more if that patient came in with cavities.”* ^*P13, 48 YO, Low referrer*^

In addition, some informants were not familiar with the most recent evidence based preventive dentistry guidelines (Delivering Better Oral Health: An evidence-based toolkit for prevention) [13]. They blamed lack of direct promotion for that, as one dentist noted:

*“No I didn’t know of this, because no leaflets or information were sent to the surgery anymore.”* ^*P17, 63 YO, Low referrer.*^

This unfamiliarity was reflected in inaccurate recommendations given to patients regarding fluoride toothpaste concentration, and variable frequency and criteria, including age and caries risk, for fluoride varnish application. For example, when asked about what toothpaste recommendations are given to children, an informant responded:

*“depends how old they are but, normally if it’s above 6 year olds and they are high risk then I tell them to use 1150 PPM just a smear of adult toothpaste and that’s it, otherwise 950 to 1000 PPM.”* ^*P9, 26 YO, Medium referrer*^

*“For under six I normally say use the kiddies’ ones, 1000 PPM”.* ^*P8, 31 YO, Medium referrer*^

There was no consensus between informants when asked about criteria and frequency of fluoride varnish application and they all gave variant responses:

*“I use it with the high risk patients that have more than five fillings, we use it every in every visit. If the patient doesn’t have any caries we never use it”.* ^*P14, 39 YO, Low referrer*^

*“Even if the children have a low decay, we tend to just put it on their teeth”.* ^*P3, 29 YO, High referrer*^

*“After drying and very sparingly, for sort of medium to high risk patients”.* ^*P8, 31 YO, Medium referrer.*^

*“I normally do it for children 6 to sort of 16 or 17”.* ^*P9, 26 YO, Medium referrer*^

Some informants were still not using fluoride varnish at all, either due to what they perceived as lack of training or lack of time and resources, interestingly, despite being low referrers.

*“I mean delivering fluoride is quite a difficult business… it’s difficult out here … We don’t really have even the time to allocate to a child”.* ^*P12, 57 YO, Low referrer.*^

In one instance, fluoride varnish was not used due to lack of belief in the evidence.

*“I don’t apply fluoride varnish, I don’t believe it in, you don’t need it”* ^*P1, 73 YO, Medium referrer.*^

Oral health advice that informants provided to those children tended to revolve around reducing intake of obvious sources of sugar (i.e. sweets), and frequency of tooth brushing. The advice doesn’t seem to be tailored to each patient. Moreover, only a few informants mentioned providing advice on tooth brushing supervision, toothpaste fluoride concentration (dose) and not rinsing after brushing:

*“Proper brushing, just take care, do not eat sweets.”* ^*P2, 32 YO, High referrer.*^

*“We usually give them like written information about sugar and oral hygiene instructions, we insist a lot about food.”* ^*P14, 39 YO, Low referrer.*^

It was interesting to note that the cohort of patients seems to be well distributed between practices. All informants, including those from high referral clinics reported that the number of children they individually refer is low. This can be of important implication on planning future strategies to improve the oral health of this cohort.

*“I think to specify actual extractions under GA would only be about five a year.”* ^*P5, 30 YO, High referrer*^

*“Overall we don’t have a high referral rate to the hospital.”* ^*P10, 49 YO, Medium referrer*^

*“Well it’s very, very rare that I have kids for general anaesthesia.”* ^*P15, 34 YO, Low referrer*^

### Inadequate secondary care communication and engagement

The informants reported issues in communication between the hospital and both referring dentists and families. Many of them found discharge letters lacking sufficient information. They reported that it will be useful, and potentially improve post-operative follow up, if more information was provided in these letters, the type of information can be divided into two categories:

(i) Information about care provided in hospital: this includes details on treatment provided and rationale:

*“It would be good to see: has this patient been seen? What has been done apart from their exodontia or filling? Have they had advice? We don’t know where we are picking it up from!”* ^*P13, 48 YO, Low referrer*^

(ii) Information regarding needed post-operative recall: Information regarding preventive care and maintenance needed following completion of treatment under general anaesthesia will be helpful:

*“I think they should also indicate the things they would like us to focus on, advice and maintenance…”* ^*P4, 37 YO, High referrer*^

One dentist explained how providing the family with information on the importance of recall after treatment under GA has been completed can give them a sense of continuity in treatment:

*“I think if the hospital emphasizes to the parents: okay we’ve done the treatment, we just want them to come to the surgery again within three weeks or whatever, they know they have to come back here again for the routine appointment”.* ^*P7, 59 YO, Medium referrer*^

The informants also suggested that more efforts to promote oral health need to be taken by the hospital upon the child’s referral. They noted that this is one of the rare chances to capture the families of those children to deliver an oral health intervention. In their opinion, those families might be more responsive to advice delivered by the hospital compared to the local dentist due to some form of perceived hierarchy.

*“Patients take what comes from the hospital as a gospel, when it comes to the practice, not necessarily, you are just a dentist.”* ^*P13, 48 YO, Low referrer*^

*“I find when you speak in the hospital to the children and parents they do listen a little bit more, and they come back to me and say I need this treatment to be followed up.”* ^*P12, 57 YO, Low referrer*^

### Failure in establishing national policy to grasp the width and depth of the problem

In an apparent call for change in wider public policies, informants noted that those children are being surrounded by an unhealthy environment, making oral health promotion at the dentist alone difficult. For example, one dentist described the large amounts of sugary drinks being promoted for children at the local store by saying:

*“There are 3 aisles of sweet drinks and it is what they (the children) are drinking”* ^*P11, 51 YO, Medium referrer*^ *.*

Informants felt isolated in their efforts to promote oral health, they noted that in order to tackle the issue, there is a need to broaden the involvement of others in primary care setting, including general practices, maternity wards, etc. In addition, a common risk factor approach can be followed, so that dentists are not isolated in their “nagging” as one informant put it:

*“I say long term the sugar is not good for their general health, obesity and other problems down the road. So I tried to give it the holistic approach, it’s not just me the dentist nagging, you will be nagged later on by the medics down the road”.* ^*P11, 51 YO, Medium referrer*^

Finally, informants demanded wider efforts to create a healthier environment for those families. Policies are needed to ensure oral health promotion starts in the community using various outlets such as media and schools before those families even step into the dental practice:

*“I think it is tricky, once you get them to come to the dentist they are more likely to come back, that’s just the first thing. So I think just general motivation and things on a broad spectrum: posters and adverts on TV and all that will obviously help”.* ^*P9, 26 YO, Medium referrer*^

In summary, there is evidence of failure on all fronts when it comes to promoting the oral health of those children and their families. GDPs are feeling frustrated with multiple challenges that hinder their oral health promotion efforts, and are left isolated without sufficient support from other elements involved in promoting the wellbeing of those children.

## Discussion

When it comes to oral health care, high caries risk children seem to be stuck in a cycle of despair; they are being failed on multiple levels by everyone responsible for their care, whether its parents, health providers, local environment and society, or system and policies. There is an undercurrent of despair and helplessness in the GDPs we’ve interviewed, who see those children when they present for the first time to their surgeries with multiple decayed teeth, in pain, and typically coming from families who, in their eyes, have poor parenting skills or simply don’t speak English or have cultural differences. As such, oral health promotion in those children needs to be multi-agency and multidimensional, and does not just rest with the GDPs. Indeed, efforts for oral health promotion may fall heaviest on policy makers.

Interviewed GDPs perceived that parents of this cohort of children frequently hold negative attitudes towards dental health, lack oral health knowledge, and are in need of parenting skills support. Previous studies suggested that parental motivation and perceived improvements in oral health are crucial factors the amount of time GDPs are willing to spend giving oral health advice [26], putting high caries risk children of parents whom GDPs perceive have poor motivation or parenting skills at even further disadvantage. Those parents might be in need of support that focuses on improving their parenting skills and attitudes towards oral health. Indeed, recent evidence suggests that parenting skills and style are closely related to children’s dietary habits as well as oral health [27,28]. Perhaps more importantly, it might be time to re-examine the paradigm and determine if GDPs need new tools to improve their communication and understanding of those families.

The GDPs also discussed how social inequalities take a toll on children’s oral health. The association between deprivation and oral health is well documented [29]. An issue that is reported in the present study, but perhaps more local to the hospital’s catchment area, is the difficulty in communication and service provision to children of immigrant families. It’s debatable whether these observations are accurate or a form of stereotyping. A previous study at KCH reported that only 15% of children referred for dental extractions under GA had lived in another country [10]. However, it may be that there was a bias in participants with immigrant parents choosing not to participate, which might explain the difference in findings. This is not a new finding in other high income countries; a previous study reported a significant difference in caries prevalence between immigrants and native population of Norway, with caries prevalence in five year old immigrants reaching 88% [30]. Furthermore, earlier research has suggested that ethnic minorities perceive barriers to dental services, including language, cultural misunderstandings, costs, and dentist mistrust [31], which might explain the infrequent attendance and late presentation of those children.

The role of GDPs in the provision of preventive care and oral health promotion cannot be ignored. A study in 2013 suggested that leading a prevention oriented primary dental care practice, where parents are invited to register children upon birth, can affect their oral health practices, including age of dental registration and oral hygiene habits [32]. Unfortunately, at the moment, efforts provided at a primary care level, especially for high risk children might be falling short. Advice provided to those families seems to be generic rather than tailored. The main focus seems to revolve around sugary foods avoidance and frequency of tooth brushing. Other researchers reported likewise [10,33]. Some interviewed GDPs also displayed a lack of familiarity with “Delivering better oral health: an evidence-based toolkit for prevention”, the most recent guideline for caries prevention [13]. A few still don’t use fluoride varnish; they cited lack of training, evidence, resources, and time as the reason for this. NHS statistics in recent years have shown an overall significant increase in the use of fluoride varnish by GDPs [34,35]. It is important to ensure that high caries risk children from challenged families are not left behind as they need it the most [34]. There is a need for better promotion of guidelines, as well as better training and support for GDPs on how these guidelines can be applied in general practice. Indeed, even though the Department of Health has provided the dentist with the evidence for preventive treatment, it has failed to support GDPs, or primary care services generally, in practically implementing these preventive measures [36].

One interesting observation in this study was that according to the interviewees, the number of children referred by each individual dentist was surprisingly small and this fitted with the hospital activity data. The difference in numbers referred from practices might simply be caused by the size of each practice or its geographical location and proximity to the secondary care GA extraction centre. On the positive side, if the practice referrals are indeed low then development of tailored oral health promotion for high risk referred families might not be as overwhelming to report or arduous to deliver as one might expect.

The interviewed GDPs suggested that secondary care providers should play a larger role in promoting those children’s oral health rather than just provide the surgical treatment under GA. Providing an oral health intervention for those children when they attend for extractions under GA might be of benefit, and can constitute the missing link in communication between families, primary and secondary care. The informants also pointed out the importance of improved communication between primary and secondary care providers. A recent audit at KCH recommended a new form of discharge letters that includes more information on recommended preventive care. This problem is not localised to our hospital only; a previous study in Yorkshire reported that over a half of these letters didn’t include demands to the dentist to provide preventive care and advice following discharge [37].

There is a need for an improved financial support for preventive dentistry, as the 2006 NHS remuneration contract system hasn’t provided the support necessary. As mentioned in the Steele report: “We therefore recommend that the activity payments have a more sensitive banding structure and less range in value and explicitly recognise preventive activity” [18]. The new ‘Steele’ pilot studies seems to provide better support for preventive dentistry and it was notable that the two dentists undertaking these were satisfied in this regard.

Finally, and perhaps most importantly, policy makers have the lead role in improving provided care and promoting oral health in those children and their families. Multiagency national level action is required to tackle the issue of dental caries in young children. This action should include an “upstream” approach that tackles the social and cultural determinants of the disease supplemented by “downstream” action focusing on oral health promotion [38]. We are living in a culture where parents and children are constantly bombarded by sugary foods and drinks, readily available and heavily marketed [39]. Promoting oral health needs in those families should start before they attend primary dental care services, by which time it is too late for many. The interviewed GDPs felt isolated; there is a need to broaden the spectrum of personnel involved in, including nurses, health visitors, medical practitioners, and school teachers. It is time step up promotion oral health promotion efforts as is recommended in the Ottawa Health Charter [40]. Public Health England has recently recommended adopting an integrated approach that includes various partners to achieve oral health improvements [41]. A further example, is the “ChildSmile” initiative in Scotland, where a collaborative approach to the delivery of oral health promotion, involving tooth brushing in nurseries and schools, fluoride varnish application, and improving access to primary dental care, has begun to improve the oral health of young children and reduce inequalities [42].

We acknowledge that this study has its limitations, the informants interviewed are all practicing in our hospital’s catchment area and the generalisability of their views might be questioned. Some of those invited didn’t agree to take part and might have different opinions. And finally, those interviewed might be providing the views they assume will be professionally or socially acceptable. Nevertheless, we strived to include dentists with different referral patterns, experience and age in this study. The interviewer travelled to the informants’ practices to perform the interviews in an environment that is comfortable and non-threatening to them. They appeared to provide forthright and candid responses and gave the impression that they wanted to do better for those children and their families but felt helpless to do so and frustrated that they could not do more than refer for tooth extraction.

## Conclusion

High caries risk children and their families are being failed on multiple levels. Improving their oral health has proven to be a complex issue that intertwines various factors related to our social, economic and political environment. In the present study, it can be concluded that GDPs in England feel frustrated and isolated, and are facing barriers that are related to the child, parents, social and cultural environment, level of training, guideline implementation, secondary care communication, health policy and funding.

The depth of the issue leaves a lot of room for improvement, and the heaviest burden falls on policy makers, who have the opportunity to promote initiatives that drive change. There is a need for a multi-agency multidimensional effort to relieve the social determinants of the disease, as well as broaden the width and depth of stakeholder involvement in oral health promotion that is acceptable to parents. In parallel, there is a need to re-evaluate how preventive dental care is funded, and how clinical guidelines can be implemented in practice. In addition, local action to promote the oral health of children referred for extraction under GA, and improve communication between primary and secondary care, needs to be investigated in a research informed manner.

## Abbreviations

COREQ: Consolidated criteria for reporting qualitative research
GA: General Anaesthesia
GDP(s): General Dental Practitioner(s)
KCH: King’s College Hospital, London
LSL: Lambeth, Southwark and Lewisham
NHS: National Health Services
UK: United Kingdom
XGA: Extractions under General Anaesthesia

## References

[1] Bagramian RA, Garcia-Godoy F, Volpe AR (2009). The global increase in dental caries. A pending public health crisis. Am J Dent.

[2] Congiu G, Campus G, Luglie PF (2014). Early Childhood Caries (ECC) prevalence and background factors: a review. Oral Health Prev Dent.

[3] Public Health England (2014). Oral health survey of three-year-old children 2013: a report on the prevalence and severity of dental decay.

[4] Davies GM, Neville JS, Rooney E, Robinson M, Jones A, Perkins C (2013). National Dental Epidemiology Programme for England: oral health survey of five-year-old children 2012: a report on the prevalence and severity of dental decay.

[5] Tickle M, Milsom KM, Blinkhorn AS (2002). Inequalities in the dental treatment provided to children: an example from the UK. Community Dent Oral Epidemiol.

[6] Telford C, Murray L, Donaldson M, O’neill C (2012). An analysis examining socio-economic variations in the provision of NHS general dental practitioner care under a fee for service contract among adolescents: Northern Ireland Longitudinal Study. Community Dent Oral Epidemiol.

[7] Health and Social Care Information Centre – HSCIC (2013). Hospital episode statistics, admitted patient care, England - 2012–13: treatment specialty.

[8] Kakaounaki E, Tahmassebi JF, Fayle SA (2011). Repeat general anaesthesia, a 6-year follow up. Int J Paediatr Dent.

[9] Macpherson LM, Pine CM, Tochel C, Burnside G, Hosey MT, Adair P (2005). Factors influencing referral of children for dental extractions under general and local anaesthesia. Community Dent Health.

[10] Olley RC, Hosey MT, Renton T, Gallagher J (2011). Why are children still having preventable extractions under general anaesthetic? A service evaluation of the views of parents of a high caries risk group of children. Br Dent J.

[11] Wilhelm L (2008). Encyclopedia of Public Health.

[12] Aljafari AK, Scambler S, Gallagher J, Hosey MT (2014). Parental views on delivering preventive advice to children referred for treatment of dental caries under general anaesthesia: a qualitative investigation. Community Dent Health.

[13] Public Health England (2014). Delivering better oral health: an evidence-based toolkit for prevention, Third Edition.

[14] Pearce M, Catleugh M (2013). Are general dental practitioners providing best practice in prevention in everyday general practice?. Prim Dent J.

[15] Witton RV, Moles DR (2013). Barriers and facilitators that influence the delivery of prevention guidance in health service dental practice: a questionnaire study of practising dentists in Southwest England. Community Dent Health.

[16] Milsom KM, Blinkhorn AS, Tickle M (2008). The incidence of dental caries in the primary molar teeth of young children receiving National Health Service funded dental care in practices in the North West of England. Br Dent J.

[17] Pine CM, Harris RV, Burnside G, Merrett MC (2006). An investigation of the relationship between untreated decayed teeth and dental sepsis in 5-year-old children. Br Dent J.

[18] Steele J, Clarke J, Wilson T, Rooney E (2009). NHS dental services in England: An independent review led by Professor Jimmy Steele.

[19] Department for Communities and Local Government (2011). English indices of deprivation 2010.

[20] Office of National Statistics (2012). 2011 Census: Country of birth, local authorities in England and Wales.

[21] Gallagher JE, Cooper DJ, Wright D (2009). Deprivation and access to dental care in a socially diverse metropolitan area. Community Dent Health.

[22] Ritchie J, Lewis J (2003). Qualitative research in practice: a guide for social science students and researchers.

[23] Tong A, Sainsbury P, Craig J (2007). Consolidated criteria for reporting qualitative research (COREQ): a 32-item checklist for interviews and focus groups. Int J Qual Health Care.

[24] Crabtree BF, Miller WL, Crabtree BF, Miller WL (1999). Clinical research: a multi-method typology and qualitative roadmap. Doing qualitative research.

[25] Gallagher JE (2012). Occupational health service specification version 20.07.2012.

[26] Threlfall AG, Hunt CM, Milsom KM, Tickle M, Blinkhorn AS (2007). Exploring factors that influence general dental practitioners when providing advice to help prevent caries in children. Br Dent J.

[27] Hooley M, Skouteris H, Boganin C, Satur J, Kilpatrick N (2012). Parental influence and the development of dental caries in children aged 0–6 years: a systematic review of the literature. J Dent.

[28] Peters J, Dollman J, Petkov J, Parletta N (2012). Associations between parenting styles and nutrition knowledge and 2-5-year-old children’s fruit, vegetable and non-core food consumption. Public Health Nutr.

[29] Locker D (2000). Deprivation and oral health: a review. Community Dent Oral Epidemiol.

[30] Skeie MS, Riordan PJ, Klock KS, Espelid I (2006). Parental risk attitudes and caries-related behaviours among immigrant and western native children in Oslo. Community Dent Oral Epidemiol.

[31] Newton JT, Thorogood N, Bhavnani V, Pitt J, Gibbons DE, Gelbier S (2001). Barriers to the use of dental services by individuals from minority ethnic communities living in the United Kingdom: findings from focus groups. Prim Dent Care.

[32] Richards W (2013). Evaluating oral health promotion activity within a general dental practice. Br Dent J.

[33] Threlfall AG, Milsom KM, Hunt CM, Tickle M, Blinkhorn AS (2007). Exploring the content of the advice provided by general dental practitioners to help prevent caries in young children. Br Dent J.

[34] NHS Prescribing and Primary Care Team, H.A.S.C.I.C (2013). NHS dental statistics for England: 2012/13.

[35] NHS Prescribing and Primary Care Team, H.A.S.C.I.C (2014). NHS dental statistics for England: 2013/14.

[36] Richards W, Toy A (2007). Improving oral health with the new dental contract. Br Dent J.

[37] Ni Chaollai A, Robertson S, Dyer TA, Balmer RC, Fayle SA (2010). An evaluation of paediatric dental general anaesthesia in Yorkshire and the Humber. Br Dent J.

[38] Marmot M (2010). Fair society, healthy lives. University College London.

[39] Rodd HD, Patel V (2005). Content analysis of children’s television advertising in relation to dental health. Br Dent J.

[40] World Health Organization (1986). Ottawa charter for health promotion: an International Conference on Health Promotion, the move towards a new public health.

[41] Public Health England (2014). Improving oral health: an evidence-informed toolkit for local authorities.

[42] Mcmahon AD, Blair Y, Mccall DR, Macpherson LM (2011). Reductions in dental decay in 3-year old children in Greater Glasgow and Clyde: repeated population inspection studies over four years. BMC Oral Health.

